# All Twenty Nails Affected: Aripiprazole-Induced Photo-Onycholysis in an 11-Year-Old Boy

**DOI:** 10.7759/cureus.88770

**Published:** 2025-07-25

**Authors:** Brian A Moreno, Christopher Kowalczyk, Martin Zaiac, Ana Duarte

**Affiliations:** 1 Dermatology, Lake Erie College of Osteopathic Medicine, Bradenton, USA; 2 Dermatology, Larkin South Miami, South Miami, USA; 3 Dermatology, Herbert Wertheim College of Medicine, Florida International University, Westchester, USA; 4 Dermatology, Greater Miami Skin and Laser Center, Miami Beach, USA; 5 Dermatology, Mount Sinai Medical Center, Miami Beach, USA; 6 Dermatology, Children's Skin Center, Nicklaus Children's Hospital, Miami, USA

**Keywords:** complex dermatology, dermatology care, dermatology case report, dermatology consult, dermatology consultation, dermatology teaching, general dermatology, paediatric dermatology, pediatric dermatology, research in dermatology

## Abstract

Photo-onycholysis is a phototoxic reaction characterized by separation of the nail plate following light exposure and medication use. While commonly associated with antibiotics and psoralens, newer agents have also been implicated. We present the case of an 11-year-old male who developed diffuse brown discoloration, onycholysis, and onychomadesis of all 20 nails one month after starting liquid aripiprazole. The pattern of involvement on sun-exposed digits, preference for open-toed footwear, and exclusion of other triggers supported a diagnosis of drug-induced photo-onycholysis. The patient was managed with topical anti-inflammatories, vinegar soaks, and strict photoprotection, and was referred for dermatologic and podiatric follow-up. This case emphasizes the need for awareness of nail phototoxicity associated with atypical antipsychotics and the importance of prompt recognition to avoid unnecessary interventions.

## Introduction

Drug-induced nail pathology encompasses a broad spectrum of presentations, including onycholysis (separation of the nail plate from the nail bed), onychomadesis (complete shedding of the nail from the matrix), melanonychia (pigment bands in the nail), and dystrophic ridging. Photo-onycholysis, or phototoxic nail separation, is a drug-induced reaction in which the nail plate detaches from the bed following light exposure. These conditions account for roughly 2% of adverse cutaneous drug reactions documented in hospital registries [1]. Photo-onycholysis is classically associated with tetracyclines, fluoroquinolones, and psoralens, but more than 40 pharmacologic classes have been implicated [1,2]. The reaction is believed to arise from drug-derived photo-excited radicals that damage the nail matrix and underlying keratinocytes, producing distal detachment that is often preceded by brown or gray discoloration [2]. Pediatric cases are uncommon yet clinically relevant, as children may be more susceptible to sun exposure during outdoor activities, and nail changes can generate substantial psychosocial distress. Accurate recognition is challenging, as the latency between exposure and nail detachment may span several weeks, and onycholysis can mimic onychomycosis, traumatic nail changes, or habit-tic deformity, leading to unnecessary testing [3]. Awareness of the medication history and a high index of suspicion are therefore critical for timely diagnosis.

Aripiprazole, a dopamine D₂ partial agonist widely prescribed for autism-related irritability, tic disorders, and pediatric mood symptoms, is generally well tolerated, with most cutaneous adverse events limited to transient rashes [4]. Nail phototoxicity attributable to aripiprazole remains exceedingly rare, with only isolated case reports describing diffuse onycholysis or onychomadesis [3,4]. Proposed mechanisms mirror those of classic photo-onycholytic agents: the drug or its metabolites absorb ultraviolet A (UVA) or visible light, generate reactive oxygen species, and subsequently disrupt matrix keratinization. Given the expanding off-label pediatric use of aripiprazole and the potential for substantial impacts on the nail unit, additional documentation of such reactions is warranted to aid clinicians in early recognition and management. We report the case of an 11-year-old male who developed pan-digit photo-onycholysis with onychomadesis of all 10 toes shortly after starting liquid aripiprazole, highlighting diagnostic clues and therapeutic considerations for this under-recognized adverse effect.

## Case presentation

**Figure 1:**
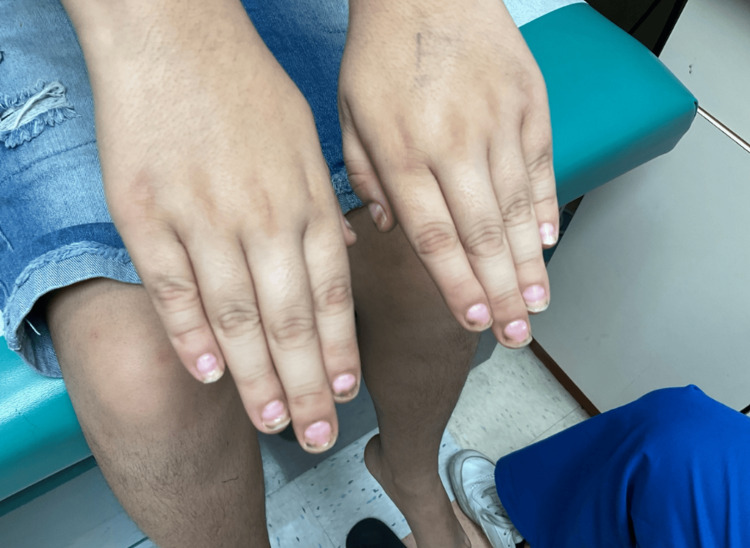
Diffuse brown discoloration of the fingernails with associated splitting, ridging, and brittleness.

**Figure 2:**
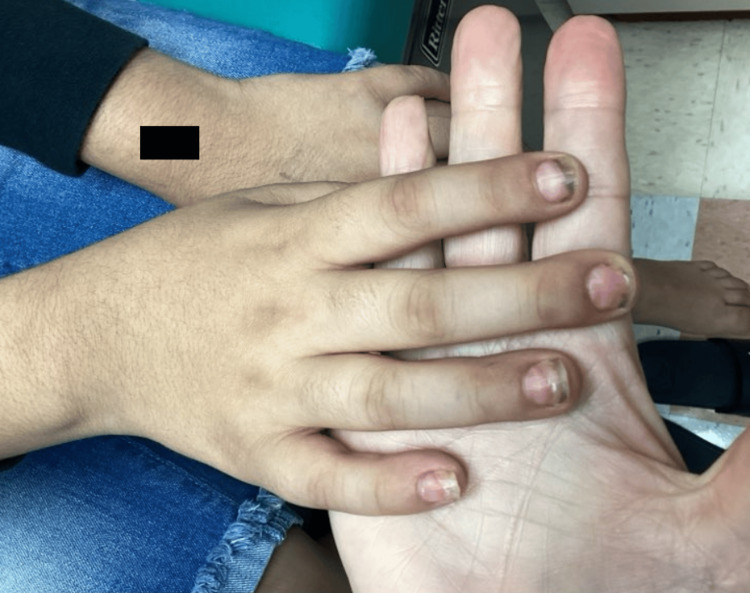
Diffuse brown discoloration of the fingernails, along with splitting, ridging, and brittleness.

An 11‑year‑old male with autism spectrum disorder (ASD), anxiety, and gastroesophageal reflux disease presented for evaluation of progressive nail changes affecting all 20 digits. His chronic medications included clonidine 0.2 mg oral tablet nightly, trazodone 50 mg oral tablet nightly, and aripiprazole 1 mg/mL oral solution (1 mL daily), which was initiated one month prior; no new drugs, supplements, or traumatic events were reported. The patient’s mother noted a history of diffuse brown discoloration of the fingernails and toenails, followed by progressive splitting, ridging, and brittleness starting one month ago (Figures 1-2). The changes were painless but cosmetically distressing and appeared to worsen despite routine hygiene. The patient and his mother denied photosensitivity, systemic symptoms, or prior similar episodes. Family history was negative for inflammatory dermatoses, onychodystrophy, or autoimmune disease.

**Figure 3:**
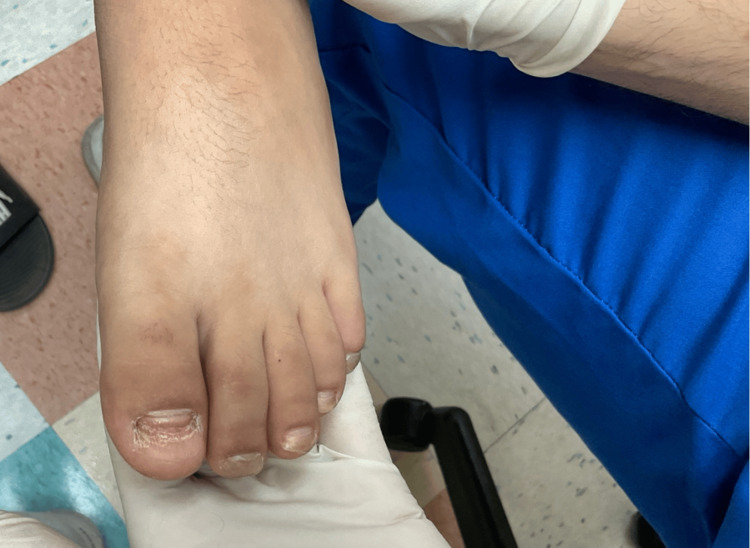
Complete onychomadesis of the left great toenail, partial onychomadesis of the remaining toes, and marked distal onycholysis with associated longitudinal ridging and lamellar splitting.

**Figure 4:**
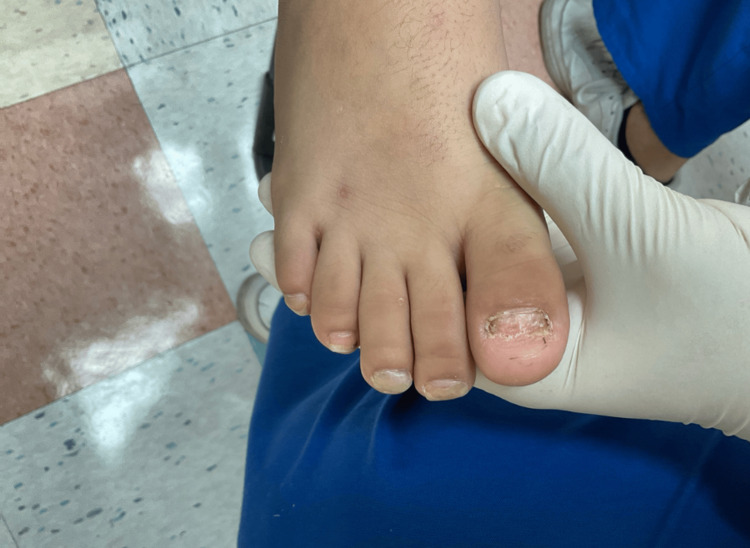
Complete onychomadesis of the right great toenail, partial onychomadesis of the remaining toes, and marked distal onycholysis with longitudinal ridging and lamellar splitting.

Physical examination revealed a well‑nourished, developmentally appropriate male in no acute distress. Dermatologic inspection demonstrated complete onychomadesis of both great toenails, partial onychomadesis of the remaining toes, and marked distal onycholysis with longitudinal ridging and lamellar splitting of the remaining fingernails and toenails (Figures 3-4). Although the discoloration appeared grossly as pigment, dermoscopic inspection revealed it to be subungual debris rather than melanin. Periungual skin was intact, without erythema, scale, or pustulation. A solitary 3‑mm light‑brown macule over the left clavicular neck was noted but appeared clinically benign. No mucosal involvement, alopecia, or cutaneous lesions suggestive of psoriasis or lichen planus were present.

The patient’s mother was again asked about the relationship between initiating aripiprazole and the nail changes, and she readily agreed that the latter closely followed the former. Additionally, we asked about the patient’s preference in footwear, as he was wearing open-toed, slide-on sandals in clinic, and they both confirmed he most prefers these sandals over other footwear.

Given the temporal relationship to aripiprazole exposure and onset of nail changes, the distribution on photo‑exposed digits, preferred footwear, and the absence of concomitant dermatoses, drug‑induced photo-onycholysis, likely from aripiprazole, was favored. Differential diagnoses of idiopathic onychomadesis, fungal onychodystrophy, and trauma were considered but believed to be less likely. The patient was counseled on nail protection and prescribed topical mometasone 0.1% solution (morning) and calcipotriene 0.005% solution (evening) to reduce inflammation and promote regrowth. Daily 1:1 vinegar‑water soaks were recommended to debride subungual debris, with specific instructions to avoid instrumentation around or beneath the nails. The primary team arranged pediatric podiatry follow‑up and communicated with a consulting dermatologist and nail expert, who concurred with the presumptive diagnosis. Plans included monitoring for spontaneous nail‐plate regeneration and reassessing the risk-benefit ratio of continuing aripiprazole should progression occur. At the time of documentation, the patient had not yet followed up with dermatology or psychiatry. Given the complexity of his behavioral health needs and our limited insight into his psychiatric history, we advised deferring any changes to aripiprazole until further evaluation by his prescribing psychiatrist. Fungal testing, including potassium hydroxide (KOH) preparation or nail culture, was not pursued, as the diffuse, simultaneous onset across all 20 nails with a clear temporal association to sun exposure and aripiprazole initiation made onychomycosis clinically unlikely.

## Discussion

Photo-onycholysis represents a phototoxic injury in which drug‑derived photosensitizers absorb UVA or visible light, generate reactive oxygen species, and damage nail‑matrix keratinocytes, ultimately separating the nail plate from its bed [1,2]. More than forty pharmacologic classes have been implicated, but only a handful of atypical antipsychotics appear in comprehensive reviews [1]. Aripiprazole‑associated nail toxicity is particularly rare; published reports describe isolated or oligodactylous involvement, often after dose escalation or formulation change [3,4]. Our patient extends this spectrum by demonstrating pan‑digit onycholysis and onychomadesis within a month of starting a low‑dose liquid formulation, suggesting that even minimal systemic exposure can trigger phototoxicity when combined with intense outdoor activity.

The absence of periungual inflammation or systemic symptoms, coupled with involvement of all nails in the classic distal detachment pattern, aligns with photo-onycholysis rather than onychomycosis, nail psoriasis, or trauma [2]. Although clonidine and trazodone were co‑administered, neither drug has been linked to phototoxic nail changes, reinforcing the causal association with aripiprazole [1]. Based on the temporal relationship, clinical pattern, and absence of alternative causes, this adverse reaction would be considered ‘probable’ under the Naranjo Adverse Drug Reaction Probability Scale [4]. This case therefore adds weight to the hypothesis that photo‑activated aripiprazole metabolites can act directly on the nail matrix, even in pediatric patients without chronic medication exposure [4].

Management of drug‑induced photo-onycholysis is largely supportive and centers on eliminating the inciting agent, protecting nails from further photodamage, and controlling secondary inflammation. Discontinuation or dose reduction of the suspected photosensitizer is considered first‑line; when psychiatric history is complex or continuity of care is limited, as in this case, continuation may be considered while arranging close multidisciplinary follow-up [2,4]. Topical anti‑inflammatory agents such as mometasone and nail‑targeted keratinocyte modulators like calcipotriene, as prescribed for our patient, may reduce matrix inflammation and expedite orderly regrowth, though high‑level evidence is lacking [1]. Vinegar soaks offer a simple, low‑cost adjunct to debride subungual debris and lower pH, thereby discouraging instrumentation and exacerbatory traumatic changes leading to secondary bacterial or fungal colonization [2].

Follow‑up every three to six months is recommended, as full nail‑plate regeneration in children often requires nine to twelve months, and persistent dystrophy may signal an alternative diagnosis such as idiopathic onychomadesis. In pediatric patients, complete nail-plate regrowth may take 9-12 months following discontinuation of the inciting agent, although partial improvement may be noted within the first 3 months [3]. Clinicians should also counsel families on the psychosocial impact of nail disfigurement and the likelihood of complete recovery once the offending medication is addressed. Documenting nail-related adverse effects in pharmacovigilance databases will help refine risk estimates for atypical antipsychotics, especially as their use rises in pediatric populations. This may ultimately inform safer prescribing practices and identify opportunities for preventive strategies such as UVA filtering or formulation adjustments. Finally, inclusion of nail adverse events in pharmacovigilance databases will refine risk estimates and inform prescribers as atypical antipsychotic use expands in pediatric populations [4].

## Conclusions

Aripiprazole-induced photo-onycholysis is a rare but recognizable adverse effect that can involve all 20 nails and mimic infectious or traumatic nail disorders. Clinicians should remain vigilant for abrupt, polydactylous nail detachment following medication changes, particularly in sun-exposed digits, and carefully review recent drug histories when evaluating onycholysis. Prompt recognition can prevent unnecessary testing, reduce morbidity, and support timely implementation of photoprotection and anti-inflammatory therapy. As pediatric prescriptions for atypical antipsychotics continue to rise, heightened awareness of potential nail phototoxicity is essential for comprehensive patient care.
